# DTi2Vec: Drug–target interaction prediction using network embedding and ensemble learning

**DOI:** 10.1186/s13321-021-00552-w

**Published:** 2021-09-22

**Authors:** Maha A. Thafar, Rawan S. Olayan, Somayah Albaradei, Vladimir B. Bajic, Takashi Gojobori, Magbubah Essack, Xin Gao

**Affiliations:** 1 Computer, Electrical and Mathematical Sciences and Engineering Division (CEMSE), Computational Bioscience Research Center, Computer (CBRC), King Abdullah University of Science and Technology (KAUST), Thuwal, Kingdom of Saudi Arabia; 2 College of Computers and Information Technology, Computer Science Department, Taif University, Taif, Kingdom of Saudi Arabia; 3 The Jackson Laboratory for Genomic Medicine, Farmington, CT USA; 4 Faculty of Computing and Information Technology, King Abdulaziz University, Jeddah, Kingdom of Saudi Arabia

**Keywords:** Drug repositioning, Drug–target interaction, Heterogeneous network, Network embedding, Random walk, Link prediction, Representation learning, Ensemble learning, Cheminformatics

## Abstract

Drug–target interaction (DTI) prediction is a crucial step in drug discovery and repositioning as it reduces experimental validation costs if done right. Thus, developing *in-silico* methods to predict potential DTI has become a competitive research niche, with one of its main focuses being improving the prediction accuracy. Using machine learning (ML) models for this task, specifically network-based approaches, is effective and has shown great advantages over the other computational methods. However, ML model development involves upstream hand-crafted feature extraction and other processes that impact prediction accuracy. Thus, network-based representation learning techniques that provide automated feature extraction combined with traditional ML classifiers dealing with downstream link prediction tasks may be better-suited paradigms. Here, we present such a method, DTi2Vec, which identifies DTIs using network representation learning and ensemble learning techniques. DTi2Vec constructs the heterogeneous network, and then it automatically generates features for each drug and target using the nodes embedding technique. DTi2Vec demonstrated its ability in drug–target link prediction compared to several state-of-the-art network-based methods, using four benchmark datasets and large-scale data compiled from DrugBank. DTi2Vec showed a statistically significant increase in the prediction performances in terms of AUPR. We verified the "novel" predicted DTIs using several databases and scientific literature. DTi2Vec is a simple yet effective method that provides high DTI prediction performance while being scalable and efficient in computation, translating into a powerful drug repositioning tool.

**Supplementary Information:**

The online version contains supplementary material available at 10.1186/s13321-021-00552-w.

## Introduction

Identifying novel drug–target interactions (DTIs) is crucial to various biomedical and polypharmacology applications, such as drug discovery, drug repositioning [1], drug resistance, and side-effect prediction [2]. However, high experimental validation costs plague its success. Thus, before pursuing costly practical endeavors, more research efforts are now directed towards computationally predicting the more feasible DTIs first. Identifying these feasible DTIs can be ascertained in different ways (i.e., using various DTIs prediction tasks) such as via (1) determining if a drug interacts with the target or not (i.e., binary classification) [3, 4], (2) predicting the drugs' binding affinity towards the target protein (i.e., regression) [5, 6], or (3) predicting if the drug inhibits or enhances the reaction that occurs in the cell when the target is bound [7].

Several methods have been developed based on these various DTI prediction tasks. According to recent reviews focused on DTIs prediction methods [4, 8–12], we can classify such prediction methods into three main categories, namely: docking-based approaches [13–15], ligand-based approaches [16, 17], and chemogenomic-based approaches [4, 8]. Of these approaches, the go-to approach is chemogenomic-based [10, 18]. The chemogenomic-based approaches overcome the limitations of docking-based approaches (3D structure information for many target proteins are unavailable) and ligand-based approaches (the number of known ligands is limited or few), utilizing the chemical and genomic information of drugs and the target proteins instead. The chemogenomic-based approaches incorporate DTI prediction models based on network-, machine learning (ML)-, and deep learning (DL)-based methods. The ML-based methods use a feature-based approach that represents each drug–target pair by hand-crafted extracted features/feature vectors (FV) [8, 10, 19], or a similarity-based approach built based on the "guilt-by-association" hypothesis that similar drugs tend to interact with similar target proteins and vice versa [20, 21]. DL-based methods are a special type of ML that can learn representations of data with multiple abstraction levels and proved its efficiency in the biomedical domain [22, 23], including DTIs prediction [24].

This article focuses on the network-based methods; specifically, the network embedding-based methods, since our method, DTi2Vec, and the compared state-of-the-art methods belong to this category. Network-based methods [9] have been developed by formulating the prediction of DTIs as a link prediction problem in a heterogeneous graph where the goal is to uncover a novel interaction or link between drugs and targets. The network-based methods include traditional graph-based methods [9, 25–29] and network embedding methods. Traditional network-based methods predict DTIs through bipartite local model (BLM) [30, 31], network-based inference (NBI) model [32, 33], path score model (PSM) [27, 28, 34], and a special type of network-based method that are knowledge graph-based [35]. These traditional network-based methods proved their efficiency in solving DTIs link prediction. For example, DDR [27], a PSM-based method, proved its efficiency in predicting novel DTIs. It constructs a heterogeneous graph consisting of known DTIs, multiple drug–drug similarities, and multiple target–target similarities. Similarity selection and similarity integration algorithms select a subset of the similarities then fuse them using a nonlinear function (SNF) [44]. The path score for different path categories is generated as features, and then these features are fed into the RF classifier for prediction. However, such methods suffer from some limitations. They require a lot of network analysis and can only leverage from a specific subset of statistics and ML techniques due to challenges associated with handling the sparsity and high dimensionality of heterogeneous DTI networks. Thus, network-embedding methods [36–39] have emerged as a new efficient and promising paradigm to address traditional network analysis limitations. Network embedding converts the graph into low-dimensional space while preserving the network's structural and topological information maximally. Compressed yet informative FV represents the DTIs in the embedding space, and downstream link prediction tasks can be done using ML or DL classifiers. Categories of network embedding-based methods include the random walk-based method [40], matrix factorization (MF) based methods [41], and neural network (NN) based methods [42]. DNILMF [43] **(**Dual Network Integrated Logistic Matrix Factorization) is an MF-based method that integrates different similarity measures for both drugs and targets by applying a nonlinear similarity fusion technique based on the similarity network fusion method (SNF) [44]. Then it used this final combined measure to predict DTIs based on their graph neighbors. NRLMF [45] (Neighborhood Regularized Logistic Matrix Factorization) is another MF state-of-the-art method that integrated logistic MF with neighborhood regularization for DTI prediction by modeling the DTI probability for each drug–target pair using logistic MF. Next, it extracted drug-specific and target-specific latent feature vectors that represented drug and target properties. It also includes the local structure of DTIs to improve the interaction and use the drug similarities and target similarities in terms of nearest neighbors to eliminate the noise from using all similar neighbors. TriModel [37] (a very recent network-based method) utilizes a knowledge graph (KG) constructed from integrating different information sources to generate KG embeddings for entities (i.e., nodes) and relations (i.e., edges). TriModel predicted novel DTIs based on their interaction scores calculated using trained tensor factorization applied on the knowledge graph embeddings. The last state-of-the-art method worth mentioning is DTiGEMS+ [28], a path-score-based method developed to predict DTIs using graph embedding, graph mining, and similarity-based techniques. First, DTiGEMS+ integrated multiple drug–drug similarities using similarity network fusion algorithm (SNF) [4], and did the same on multiple target–target after applying a similarity selection procedure. After that, it constructed the final heterogeneous graph by augmenting the known DTIs graph with these two sub-graphs of drug–drug similarity and target–target similarity. Finally, DTiGEMS+ calculated the path score features from the full graph and fed them to the ML classifier.

Current methods that address the DTI prediction problem still suffer from high false-positive rates that need improvement. Thus, in this study, DTi2Vec addresses the DTI link prediction problem in a heterogeneous network using graph embedding and ensemble learning techniques. Figure 2 provides an overview of the pipeline used in our method. The DTi2Vec method's objective is to predict novel DTIs with high accuracy and avoid the limitations associated with literature methods of traditional network analysis. We performed an empirical evaluation by comparing DTI prediction performance of the proposed method DTi2Vec to several network-based state-of-the-art methods using four benchmark datasets and a large-scale FDA_DrugBank dataset. We demonstrated our approach's effectiveness in terms of the AUPR evaluation metric and error reduction rate, and verified novel DTIs predicted by DTi2Vec using reliable databases and scientific literature.

## Materials and method

### DTIs datasets

We used five datasets for this work, of which four were the "gold standard" Yamanishi_08 [46] datasets. The Yamanishi datasets are families of target proteins (http://web.kuicr.kyoto-u.ac.jp/supp/yoshi/drugtarget/), including (1) G protein-coupled receptors (GPCR), (2) ion channels (IC), (3) nuclear receptors (NR), and (4) enzymes (E). Yamanishi et al. [46] collated the DTI data from several reliable sources, namely KEGG BRITE [47, 48], BRENDA [49], SuperTarget [50], and DrugBank [51] databases released in 2008. They also collated the chemical structures of drugs/compounds from the KEGG LIGAND and KEGG DRUG databases [47, 48]. They then calculated the similarity scores representing drug features for each pair of drugs using [52] by finding the common chemical substructure. They further collected the targets' amino acid sequences from the KEGG GENES database [47, 48], then calculated the sequence similarities using the normalized Smith-Waterman scores [53] based on the alignment of protein sequences.

The fifth dataset, FDA_DrugBank, used to evaluate DDR [27] and TriModel [37] methods, was retrieved from the 5.0.3 version of the DrugBank database [51]. The FDA_DrugBank dataset only includes the DTIs with Food and Drug Administration (FDA) approval (https://www.drugbank.ca). We calculated drug similarity SIMCOMP scores, as well as normalized Smith-Waterman scores for the targets.

Table 1 summarizes the statistics of the datasets used in our study. The sparsity ratio is the number of known DTIs divided by the number of unknown DTIs. It reflects that the data is highly imbalanced between negative and positive samples (Table 1).

**Table 1:** Benchmark Yamanishi_08 datasets and FDA_DrugBank dataset statistics

Statistics	Benchmark datasets				FDA_DrugBank
	NR	GPCR	IC	Enzyme	
Number of drugs	54	223	210	445	1,482
Number of targets	26	95	204	664	1,408
Known DTIs	90	635	1476	2926	9881
Unknown DTIs	1314	20,550	41,364	292,554	2,076,775
Sparsity ratio	0.068	0.031	0.036	0.010	0.005

**Table 2:** Fusion functions for learning drug–target edge representations

Fusion function	Function equation
1. Concatenation	$\left[ { f\left( d \right) + f\left( t \right) } \right] = f\left( d \right) f\left( t \right)$
2. Hadamard	[*f*(*d*) * f*(*t*)]$= f\left( d \right) * f\left( t \right)$
3. Average	[*f*(*d*) * f*(*t*)] $= \left( {f\left( d \right) + f\left( t \right)} \right)/2$
4. Weighted-L1	$\left\| { f\left( d \right) . f\left( t \right)} \right\|_{1} = \left| {f\left( d \right) - f\left( t \right)} \right|$
5. Weighted-L2	$\left\| {f\left( d \right) . f\left( t \right)} \right\|_{2} = \left| {f\left( d \right) - f\left( t \right)} \right|^{2}$

represents element wise matrix multiplication, and  represents the average of the two matrices

### Link prediction formulation in the heterogeneous network

**Fig. 1:**
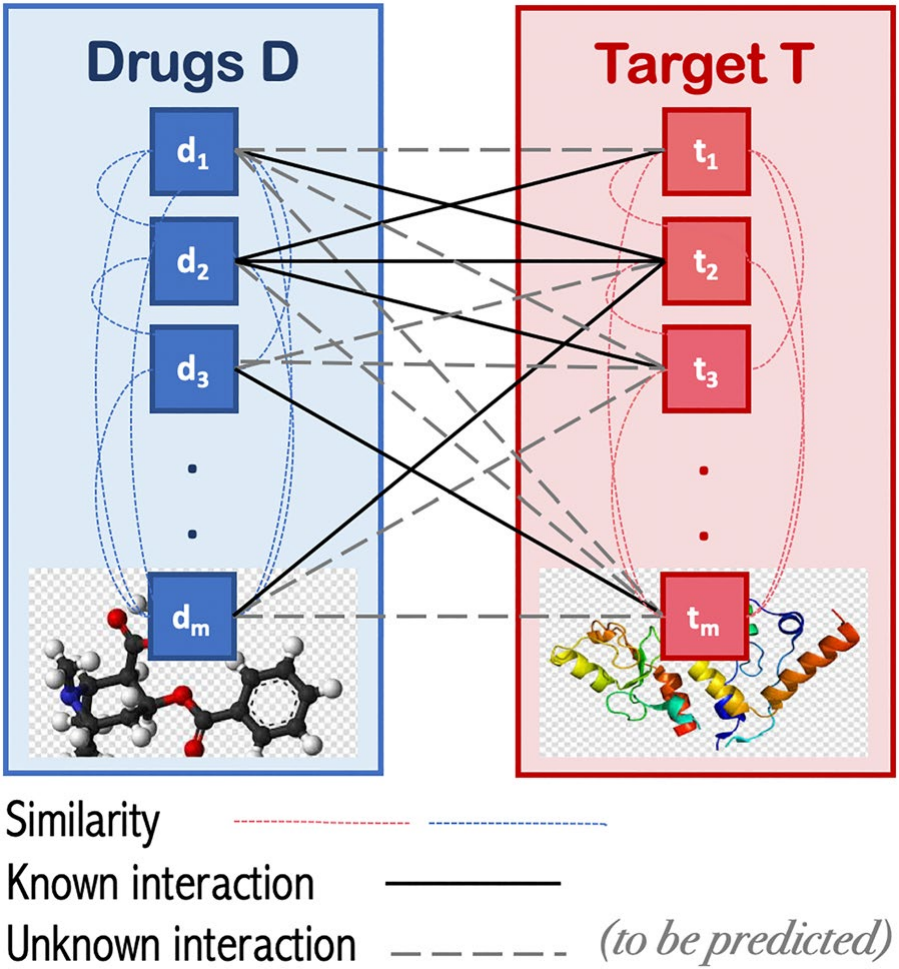
An illustration of the underlying DTI link prediction paradigm

DTI prediction was formulated as a link prediction problem in a heterogeneous graph. The goal is to predict the likelihood of a missing link (i.e., edge) between drug–target nodes in the DTI network, as shown in Fig. 1. We constructed a heterogeneous graph by defining and then augmenting all three networks (the DTIs network, drug–drug similarity network, and target-target similarity network). Thus, we obtained weighted heterogeneous graph *G (V, E)* that consists of a set of drugs *D* = {*d*_*1*_*, d*_*2*_*, …, d*_*m*_} and a set of targets *T* = {*t*_*1*_*, t*_*2*_*, …, t*_*n*_}, where *m* is the number of drugs and *n* is the number of targets, respectively. Before augmenting each similarity network, we filtered each network separately by applying the K nearest neighbor (KNN) algorithm to keep the top k similar nodes as described later in the preprocessing section. In the heterogeneous graph *G*, drug–target edges represent the known DTIs and have weight = 1, and the drug–drug (or target-target) edges represent the similarity scores and have the weight range of (0, 1]. We defined the DTIs as an m * n adjacency matrix Y that specify the class labels between each drug–target pair as follow:

1 $${y}_{ij}=\left\{\begin{array}{c}1, if there is an interaction between {d}_{i} and {t}_{j}\\ 0, otherwise.\end{array}\right.$$

where *y*_*ij*_ corresponds to the < *i, j* > element of matrix *Y*. The samples with class labels = 0, called negative samples (i.e., unknown interaction), are constructed by generating an edge between all possible drug–target pairs with no edge connecting them (i.e., unknown interaction). This process reflects a real-life scenario where negative samples are much larger than positive ones. Then, *X* vector is constructed, where *X* = *{x*_*1*_*, x*_*2*_*, …, x*_n*m_*}*, to represent each data sample (i.e., drug–target pairs feature vector) as will be described later.

### Workflow of the DTi2Vec model

**Fig. 2:**
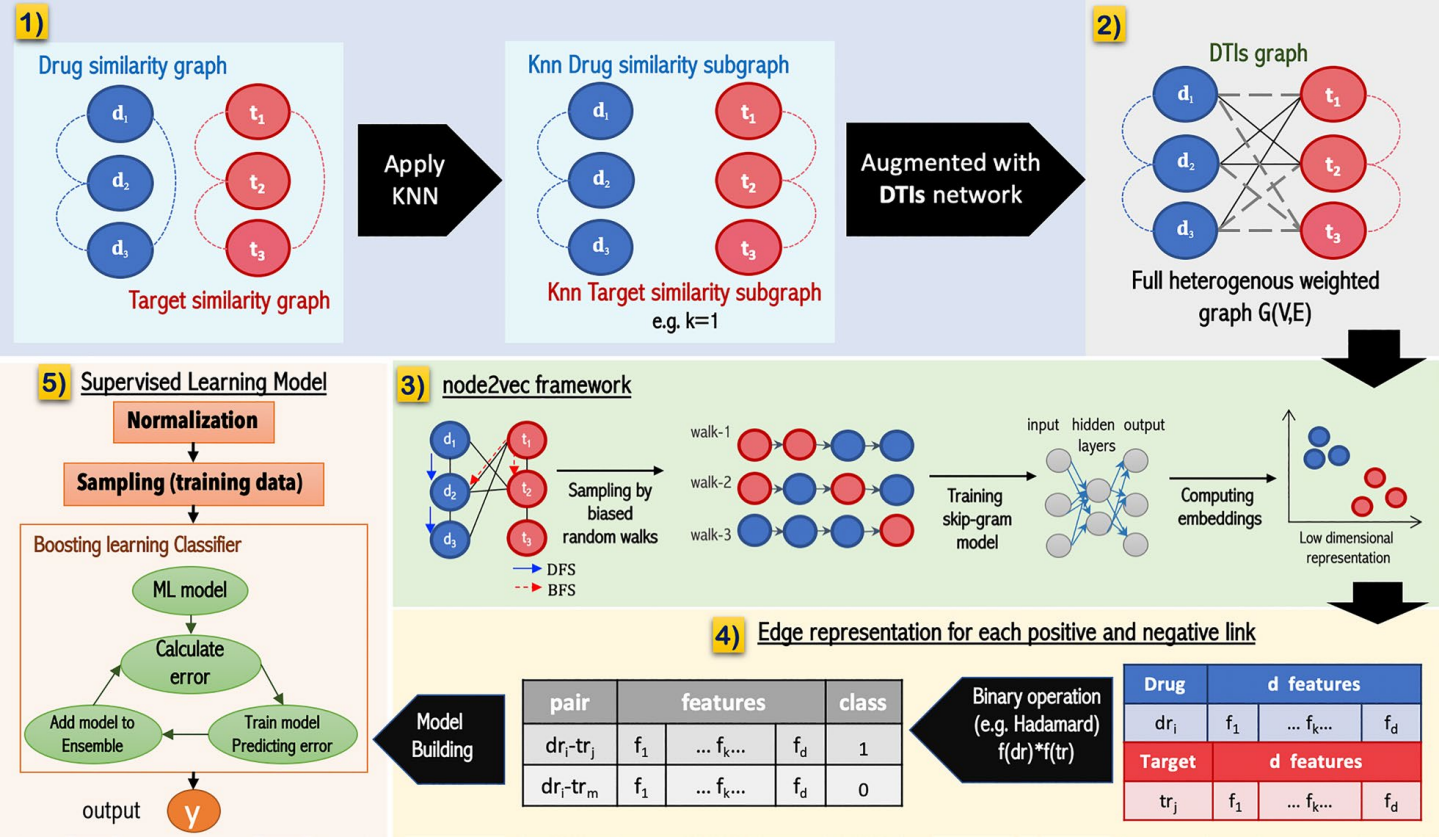
DTi2Vec Method Flowchart, (1) Filter the TTsim and DDsim graphs, (2) Construct a full DTI network by augmenting the three graphs, (3) Apply the three-step node2vec framework on the full DTI network, (4) Generate edge representation for each drug-target pair, (5) Feed the feature vector (FV) into ensemble boosting classifier to output the class labels

In Fig. 2, we show that implementing DTi2Vec involves five main steps, which are:

1. Preprocessing the drug similarity and target similarity networks.
2. Constructing a heterogeneous network *G (V,E)* by augmenting the DTI graph (training part) with the k-nearest neighbor drugs subgraph (KNN-DDsim) and the k-nearest neighbor targets subgraph (KNN-TTsim).
3. Applying a biased random walk using node2vec framework to generate feature representation (i.e., embedding) for each node (drug and target).
4. Creating drug–target edge embeddings by generating embeddings for each drug and each target and then combining them using several fusion functions.
5. Classifying the data samples by ensemble learning using boosting classifiers to predict the probability scores for each class.

We provide a detailed explanation of each step below.

### Preprocessing

**Fig. 3:**
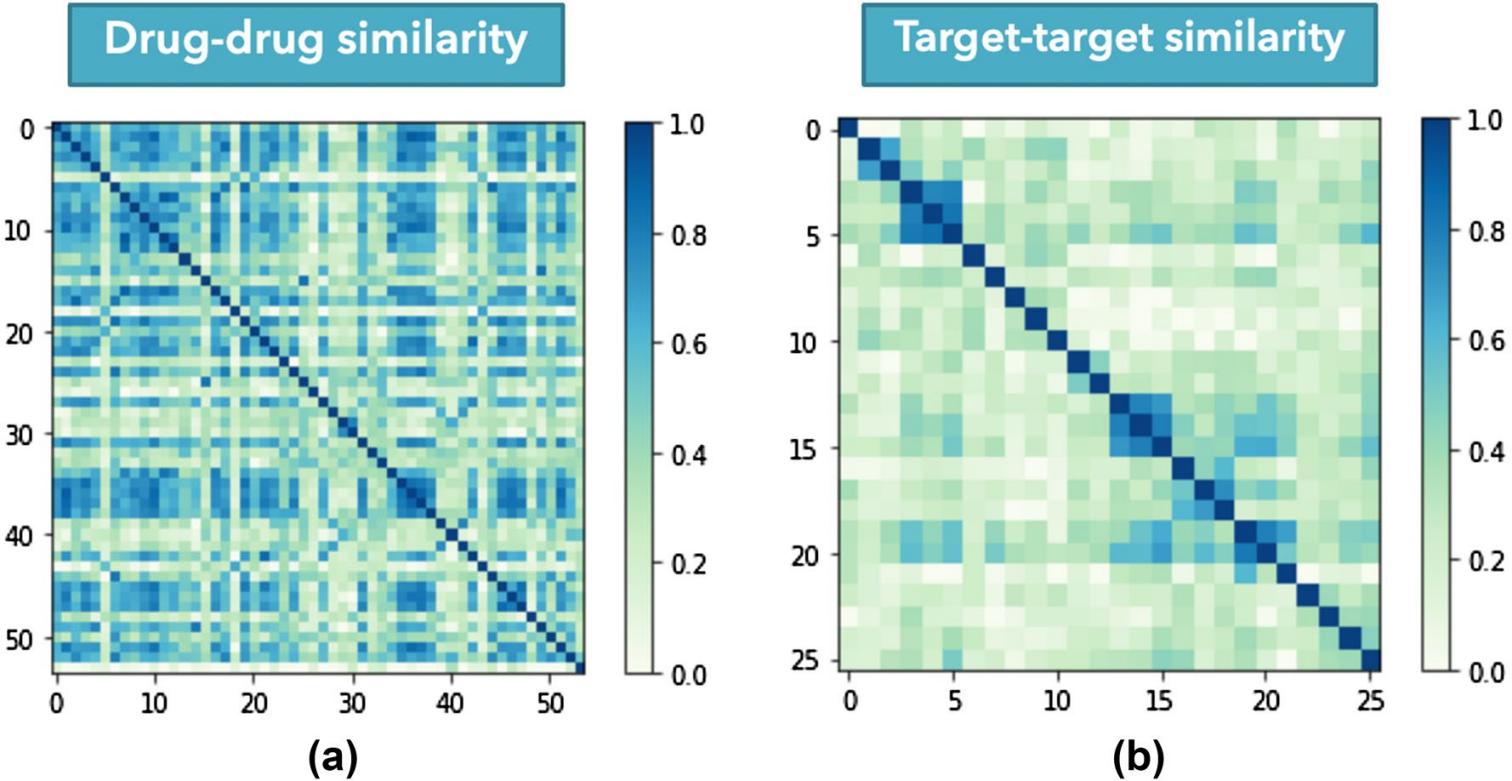
Visualization of similarity matrices of the NR dataset, **a** drug-drug similarity matrix, **b** target–target similarity matrix

When the DTI graph includes the similarity scores as edges connecting similar drugs or similar targets, the graph becomes a complex network, especially when dealing with a huge number of drugs or targets. Several similarity scores were very low or shallow, not providing any informative meaning (see the drug–drug similarity and target-target similarity matrices for the NR dataset in Fig. 3). Thus, instead of using all similarity edges for the drug similarity graph, we ranked all similarity scores for each drug in descending order and removed all the drugs except the top-k similar drugs as per the k-nearest neighbors (KNN) algorithm. Finally, we applied the same process to the target similarity network and kept the KNN determined top-k similar targets for each target node. Through this process, we generated a KNN drugs similarity subgraph and a KNN targets similarity subgraph, augmented with the training part of DTIs, as shown in Fig. 2.1. Choosing the k value is not an easy task. If the k setting is too large, it increases the time and memory complexity, making the process computationally expensive. Still, if the k setting is too small, some useful information could be dropped and affect the performance. Thus, we conducted several experiments to find the optimal number of nearest neighbors k, using different values of k. We apply this optimization process by assigning various values to k, comparing the model performance under each k value in terms of AUPR in the tenfold CV, and then selecting the k value based on the best-performing model. Using this technique means we reduced noise or meaningless information, introduced when using all similarity scores, affecting performance. It also decreases the node2vec model’s time and memory complexity since the number of the edges for each similarity network is reduced from *m (m − 1) /2* to (k*m), where *m* is the number of the nodes.

### Network embedding technique

Our study applied the graph embedding technique to transform nodes, edges, and features (i.e., similarity) into vector space by converting the graph into a low dimensional space while maximally preserving graph structure, nodes’ relationship, and other relevant properties. In the following subsections, we explain in more detail one of the random walk-based embedding techniques, the benefit of using this technique, how it works, and how we generate the feature vector for each drug–target pair.

#### Random walk-based embedding for features generation

A random walk is a stochastic process of graph traversing to describe node sequences (i.e., path) consisting of steps selected uniformly among the present vertex neighbors. Several graph embedding techniques have been developed based on utilizing random walk in the heterogeneous networks to improve the quality of feature learning (i.e., embedding) [54, 55].

In our work, we applied node2vec [56], a semi-supervised feature representation learning technique for nodes in a network that optimizes the objective of preserving the neighborhood. Node2vec is a generalized version of DeepWalk [57] inspired by the famous natural language processing (NLP) technique (word2vec) that treats the sequence of the nodes generated by a short random walk as a sentence and implements Skip-gram [58] to learn features for each node (similar to word). Skip-gram is a language model that consists of a neural network with one hidden layer. This model maximizes the co-occurrence probability among the words appearing within a window in a sentence and then predicts the neighboring words in the sentence.

For this study, we were motivated to apply the node2vec technique instead of DeepWalk because node2vec provides a biased and more flexible random walk strategy that encodes global and local proximities in the sampled path. We achieved this by interpolating two extreme searching strategies: breadth-first search (BFS) and depth-first search (DFS). BFS captures the structural equivalence characteristic while DFS discovers the graph homophily characteristics (i.e., graph communities where nodes are connected close to each other). Two hyper-parameters control which search strategy to follow: *Return parameter, p* and *In–out parameter, q.* Parameter *p* controls the likelihood of immediately revisiting a node in the walk. In contrast, parameter q controls the search's probability to differentiate between moving inward (i.e., revisiting a node) or outward to nodes that move deeper. [56] provides more detailed information about the node2vec algorithm.

In our method DTi2Vec, after we constructed the full heterogeneous weighted graph *G* = *(V, E)* consisting of known DTIs, KNN drug–drug similarity subgraph, and KNN target-target similarity subgraph, we applied the three-step node2vec framework (see Fig. 2.3). The node2vec framework translated each node in the graph *G* to a low dimensional vector space *Rd,* using a mapping function:

2 $$f:~V~ \to {R_d},~where~d\, < \,\left| v \right|$$

while preserving the graph structure and some sort of node similarity based on the network topology, resulting in obtaining a FV representation (i.e., latent feature vectors) for each drug and target nodes. Moreover, since more properties encoded by the embedder leads to better results in the downstream task performance, we utilized several node2vec hyperparameters to improve the feature representations' quality. Thus, we performed a grid search on multiple parameters to identify those values that provide the best performance using the training data during the tenfold CV process for each dataset separately. We created a dictionary of all the different hyperparameter values for the grid search, which feeds all combinations through the model for testing. We applied GridSearchCV by first initializing the node2vec hyperparameters’ values from the dictionary, then using tenfold CV to obtain the model performance using the training data. Then, a second-round is started by changing the hyperparameters' values from the dictionary and applying the same steps. Once all the values are tested, the grid search cycle finishes. The hyperparameter combinations that give the best performance during the training stage are selected. We applied this GridSearchCV procedure on each dataset separately except for the DrugBank dataset, where we used RandomizedSearchCV to tune the hyperparameters. Running these experiments on the huge DrugBank dataset will require excessive run time and large memory if GridSearchCV is applied. Therefore, we applied these two algorithms to identify the optimal values for the different datasets’ hyperparameters. This allowed us to identify the optimal values of these parameters, including embedding dimension d (i.e., the feature representation length); walk-length, the number of walks per source node; the number of the walk num-walk; and the worker number that scales and parallelizes the walks from each source node, which speeds up the feature generation process. Additional file 1: Table S1 provides the tested parameter values and indicates the optimal parameter values for each dataset.

#### Edge representation learning

After the node2vec framework generated an embedding matrix f of size *|V | x d (V is the number of vertices and d is the feature dimension),* we extracted the embedding (i.e., FV representation) for each drug and each target in the DTI network. Then, because our goal is to predict drugs and targets potentially linked, we created an embedding for each drug–target pair (i.e., edge). We accomplished this task by applying a fusion function between two FVs, because the fusion function combines two FVs to obtain a single FV. So, given two nodes drug *(d*_*i*_*)* and target *(t*_*j*_*) in G(V, E),* we generated edge feature representation *g(d*_*i*_*, t*_*j*_*)* by applying several fusion functions over the corresponding nodes FV representation *f(d*_*i*_*)* and *f(t*_*j*_*)* such that *g: V x V –* > *R *^*d*’^, where *d*^`^ is the representation size for the pair (d, t) (Table 2). We applied these fusion functions to any possible drug–target pair, including the unknown DTIs (i.e., when the edge does not exist between this drug and target).

Note, in the fusion function step (Fig. 2, panel 4) there is no dimensionality reduction. In all fusion function cases the FV sizes remained the same *(where d*^*`*^ = *d)*, except in the concatenate fusion function case, where the FV sizes increased *(d*^*`*^ = *2d)* since it connects the drugs’ FV and targets’ FV for each drug–target edge (i.e., by joining the feature representation *f(d*_*i*_*)* and *f(t*_*j*_*)* in one series). We specified the node FV dimension (between 32 and 128) that has been used for each dataset in Additional file 1: Table S1. Therefore, the final FV size for each edge was between 32 and 256 (note, 128 × 2 = 256, in the concatenate fusion function case).

### ML predictive model

After extracting all the drug–target pairs' features, we prepared the FVs to be fed into the ML classification model by first normalizing the training and test datasets separately using min–max normalization [59] and then oversampling the training data (explained below). We used min–max normalization [59] to ensure that all features are equally treated by the classifier where all features get transformed into a given range, which is [0,1] in our case. We applied min–max normalization as follows: first, we define the min–max normalization scalar, fit this scalar on the training data that saves the normalization parameters, and then transform the training data features. The last step is to normalize the features of the test data using the training normalization parameters by applying the scalar that was fitted.

#### Sampling techniques for imbalanced data

Table 1 shows that the number of unknown DTIs is much larger than the known DTIs in all datasets. This issue needs to be dealt with as ML classifiers face a problem when predicting based on imbalanced data, i.e., the ML models classify most test samples into the majority class when the minority class lacks information. We solved this problem by applying random oversampling [60] on the minority class (i.e., positive known DTIs) to obtain the same number of DTIs as the majority class (negative unknown DTIs) in the training data. This technique's implementation is done using an *imblearn* python package [61].

#### Boosting learning model

Boosting converts a family of weak learners into strong learners by combining weak learners to build ensembles: a more efficient learning algorithm that achieves better prediction performance. In boosting algorithms, the most common type of weak learner used is decision trees. Each decision tree decides what features are essential for the next model and tries to correct any mistake introduced by the previous model in a sequential process. Figure 2.5 provides an overview of how the boosting classifier works.

In DTi2Vec, supervised ML models are utilized based on two ensemble classifiers, Adaptive Boosting (AdaBoost) and eXtreme Gradient Boosting (XGBoost) classifiers [62], that are implemented respectively for DTIs prediction. AdaBoost is implemented using python library scikit-learn [63], and the XGBoost classifier is implemented using an optimized distributed gradient boosting library, called XGBoost [64]. AdaBoost uses decision trees as weak learners. XGBoost uses regression trees or CART (Classification and Regression Trees), which use continuous scores assigned to each leaf and then sum them up and provide the final prediction instead of having equal weight as in the decision trees.

XGBoost provides parallel tree boosting, which enhances performance in terms of speed. For each classifier, we performed hyperparameter optimization using tenfold CV on the training data, then tested the models using the test set to determine the set of optimal hyperparameters. We selected the model with the best performance in terms of AUPR and the corresponding set of hyperparameters. However, XGBoost tuned more hyperparameters than AdaBoost, such as the weighted regularization parameters (e.g., lambda and alpha), the tree construction algorithms, subsample ratio, etc. The most critical parameters for both classifiers include the number and maximum depth of trees, the learning rate, and the number of features and the function needed to attain a quality split.

The feature embeddings that are learned for each node and then generated for each edge (i.e., drug–target pair) are the input that is fed into these two boosting classifiers to predict the likelihood of their interactions. We constructed the ML prediction model by providing this FV *X* for both positive and negative data with their labels *Y*, as either known DTIs to represent positive labels or unknown DTIs that are treated as negative labels. This procedure is done for each dataset separately.

## Results and discussion

This section describes the evaluation protocols, the conducted experiments, and the results of our DTI prediction experiments using FDA_DrugBank and four benchmark datasets. We further compared the performance of our model, DTi2Vec, with select state-of-the-art methods. We also validated several novel DTIs predictions using scientific literature and 'reliable' databases.

### Evaluation metrics and protocols

Several performance metrics are used in the literature to compare the performance of binary classification methods. However, since the datasets we used are highly imbalanced, typical accuracy measures are not accurate. Thus, the area under the receiver operating characteristic (ROC) curve (AUC) [65] and the area under the precision-recall curve (AUPR) [65] are standard evaluation metrics used in DTIs prediction even though the AUC is over-optimistic when dealing with such a problem. Moreover, AUPR is considered more informative and provides better assessment, in such cases, by separating the predicted scores of known interactions from predicted scores of the unknown interaction. Thus, in our study, we mainly focus on the AUPR metric for performance evaluation. AUC and AUPR are defined and computed on the testing data. To obtain AUC and AUPR, we calculated the true positive rate (TPR) (also referred to as recall or sensitivity) and the precision [66], as shown in Eqs. 3 and 4, respectively.

3 $$Recall=TPR= TP/(TP+FN)$$

4 $$Precision=TP/(TP+FP)$$

The precision is the ability of the classifier not to label as positive a sample that is negative (i.e., the ratio of true positive (TP) over the total of TP and false positive (FP)). The recall is the classifier's ability to find all the positive samples (i.e., the ratio of TP over the total of TP and false negative (FN)). We constructed the ROC curve based on calculations using different recall and false predictive rate (FPR) values of different thresholds, then calculated the area under the ROC curve. Similarly, we constructed the AUPR curve based on calculations using different precision and recall values at different cut-offs, then calculated the area under this curve. The closer the value of AUC and AUPR is to 1, the better performance. We also calculated the error rate (ER) and the relative error rate reduction for the best performing model compared to the second-best performing model (ΔER), as defined in Eqs. 5 and 6, respectively:

5 $$ER= 1- AUPR$$

6 $$\Delta ER= ({ER}_{2} - {ER}_{1})/{ER}_{2}$$

where *ER*_*1*_ and *ER*_*2*_ are the error rate of the best and second-best methods, respectively. We evaluated the prediction performance of our method by applying a random setting. The random setting ensures that all the drugs and the targets are seen in the training data, which means every target and drug has at least one interaction in the training data. To facilitate the performance comparison on the benchmark Yamanishi_08 and FDA_DrugBank datasets with state-of-the-art methods, we followed these methods’ evaluation setup by conducting stratified tenfold CV for each dataset separately. We applied random CV setting where random pairs of (drug–target) are removed to be in the test data. Therefore, we randomly split the data into ten subsets in a stratified fashion wherein each subset must include the same percentage of negative and positive samples (i.e., enforcing the positive and negative class distributions in each fold to match the distribution in the whole data). We used this stratified strategy since the data is imbalanced because using the standard CV may result in some subsets without a positive label, which affects the performance and causes a computational error. We used nine subsets (with positive and negative samples) in each training stage to fit the model and kept the remaining subset, the testing data, to evaluate the model. We repeated this process ten times using each subset in the testing part. All results are reported based on the overall performance by averaging the model performance in the 10 test subsets. It is worth mentioning that we removed all the edges of the known DTIs, from the constructed graph G, in the test data before applying the node2vec technique.

Although the random setting, tenfold CV, is the most popular and widely used in computational methods, it will mimic the real application scenario when there are no known interactions for the new drugs. Therefore, we apply a new drug setting (i.e., leave drugs out) by splitting the data to have only part of the drugs available in the training phase, while the other known DTIs in testing contain drugs that have no known DTIs in the training data. We repeated this process ten times to simulate a tenfold CV but by holding new drugs in each round for testing and then averaging the results. We evaluated DTi2Vec using the new drug setting, but with the same node2vec hyperparameters and XGBoost classifier hyperparameters (the only classifier used for the "new drug" experiments) used for the random setting experiments. We also tested different node2vec embedding dimensions and chose the dimension with the best results.

We executed all experiments on a Linux Ubuntu machine with 112 processors, running some parts of our implementation in parallel, such as generating embedding using node2vec or in XGBoost classifier. We used python 3.7 for the implementation, importing some important libraries mentioned above in previous sections.

### DTi2Vec experiments performance

Table 3 provides the results we obtained for our experiments. For each dataset, separately, DTi2Vec was evaluated in terms of AUPR using two ensemble learning classifiers for different sets of FVs learned by applying five edge fusion functions. We calculated the standard deviations (std) to evaluate each models’ robustness. This process allowed us to test our methods' performance in multiple experiments before selecting the model with the best-obtained results. All std values reflected robustness in the performance of DTi2Vec (i.e., std < 0.05) except when using the very small NR dataset.

**Table 3:** Performance of DTi2Vec in terms of AUPR using AdaBoost and XGBoost classifiers on each dataset with multiple FVs generated by applying different edge representation functions for the random CV setting, and averageAUPR for each fusion function across all datasets for each classifier

Model	Fusion function (FF)	Yamanishi_08 datasets				FDA_DrugBank	AVG AUPR per FF
		NR	GPCR	IC	E		
*DTi2Vec* *AdaBoost*	Concatenate	0.74 *(0.145)*	0.83 *(0.039)*	0.97 *(0.010)*	0.97 *(0.01)*	0.77 *(0.014)*	0.856
	Hadamard	0.85 *(0.127)*	0.89 *(0.037)*	0.93 *(0.020)*	0.96 *(0.007)*	0.82 *(0.009)*	0.89
	Average	0.64 *(0.132)*	0.78 *(0.054)*	0.90 *(0.029)*	0.92 *(0.007)*	0.75 *(0.022)*	0.798
	Weighted L1	**0.92***(0.082)*	0.84 *(0.052)*	0.93 *(0.017)*	0.96 *(0.008)*	0.82 *(0.009)*	0.894
	Weighted L2	**0.92***(0.082)*	0.84 *(0.054)*	0.94 *(0.018)*	0.96 *(0.008)*	0.82 *(0.008)*	**0.896**
*DTi2Vec* *XGBoost*	Concatenate	0.72 *(0.118)*	0.87 *(0.039)*	**0.98***(0.0096)*	**0.98***(0.007)*	0.82 *(0.011)*	0.87
	Hadamard	0.81*(0.115)*	**0.90***(0.036)*	0.93 *(0.017)*	0.97 *(0.006)*	**0.88***(0.0087)*	0.898
	Average	0.68 *(0.142)*	0.81 *(0.042)*	0.91 *(0.024)*	0.95 *(0.009)*	0.78 *(0.020)*	0.826
	Weighted L1	0.88 *(0.107)*	0.84 *(0.055)*	0.94 *(0.014)*	0.97 *(0.006)*	0.87 *(0.009)*	0.9
	Weighted L2	0.89 *(0.103)*	0.84 *(0.055)*	0.94 *(0.014)*	0.97 *(0.006)*	0.87 *(0.009)*	**0.902**

AUPR in bold font with underline indicate the best result in each dataset, and the italic values between parentheses are the standard deviations

The results are not entirely consistent among the classifiers and FVs across all datasets (specifically NR dataset due to its excessive small size) (see Table 3). However, it shows the XGBoost classifier achieved the best performances except when using the NR dataset, with which the AdaBoost classifier obtained the best performance. The reason we obtained this result may be multifold. First, compared to the XGBoost classifier, Adaboost performs worse when including irrelevant features, and the noise is high, which may be the likely scenario when dealing with larger datasets. Thus, AdaBoost performed better in the smallest dataset NR. XGBoost is more robust since it has a regularization parameter that successfully reduces variance. Furthermore, the main advantage of XGBoost is its speed due to the implementation of parallel processing, making it significantly faster than the AdaBoost classifier.

Beyond the differences observed when using AdaBoost and XGBoost, we further observed that DTi2Vec's overall performances are drastically higher for the datasets grouped based on the target proteins family (i.e., Yamanishi_08 datasets) than for the FDA_DrugBank dataset. Also, with all FVs, both classifiers delivered better performance when dealing with the larger datasets, IC and E, than NR and GPCR (for Yamanishi_08 datasets), suggesting applying node2vec to large graphs generate high quality and more meaningful embedding.

The best results obtained for:

1. The NR dataset was from FVs generated using the WL1 and WL2 functions using AdaBoost,
2. The GPCR dataset was generated from FVs using the Hadamard function using XGBoost, and
3. IC and Enzyme datasets were generated using FVs from the Concatenate functions using XGBoost.

Thus, we did not observe any explicit link between the functions used to generate FVs and the individual performances observed with each dataset. However, the Average function obtained the worst performance for each dataset using both classifiers. Therefore, for any further analysis or optimization, we will exclude the average function. Also, using FVs from the Concatenate functions seemed to work better with the larger datasets (IC and Enzyme). Thus, for the FDA_DrugBank dataset, we expected to obtain the best result using the Concatenate function; however, we got better results overall for this more complex dataset using Hadamard with XGBoost and the second-best using the WL1 and WL2 functions with the same classifier. Using the AdaBoost classifier achieved lower performances than when using XGBoost, but the Hadamard, WL1, and WL2 functions performed the same and achieved better results overall. The results suggest Hadamard, WL1, and WL2 are the more stable functions that produce good quality FVs with the DrugBank dataset. For further analysis and to gain more insights into the impact of fusion function choices, we calculated the average performance in terms of AUPR for each fusion function across all datasets for each classifier separately.

With this average, we have a clear vision about the best function performance. The average performances have the same order across all fusion functions for both classifiers, as reflected in the last column in Table 3. Overall, Weighted-L2 gives the best performance based on average AUPR across all datasets for both classifiers. Weighted-L1 (second-best) and Hadamard (third-best) obtained average results very close to the best performing function WL2. The overall results indicate that WL2 and WL1 are the more highly stable functions and then the Hadamard function.

**Fig. 4:**
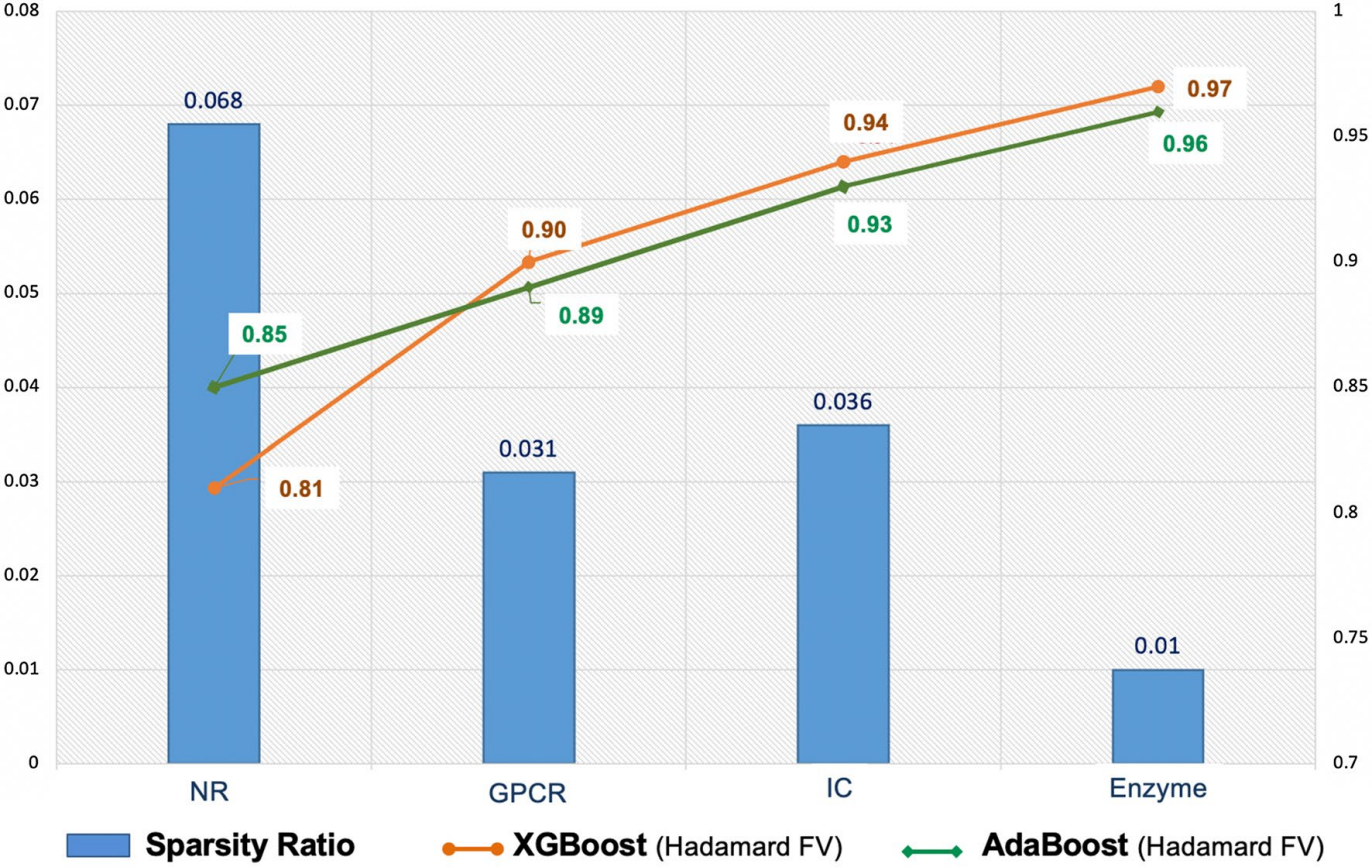
A depiction of the datasets’ sparsity ratios and the model performances in terms of AUPR applied in AdaBoost and XGBoost classifiers on Hadamard FVs

Furthermore, based on the sparsity ratio for each dataset (see Table 1) and the results of the Yamanishi_08 datasets in Table 3, we can see the link between the sparsity issue and the model performances. We observe that the model performance is low when the sparsity ratio is high, as is the case for the NR dataset. On the contrary, when the sparsity ratio decreases, the model performance increases, as seen in GPCR and Enzyme datasets. We can see that although the model has better performance using IC, the sparsity ratio is larger than for the GPCR dataset. The number of known interactions in the IC dataset being much larger than in GPCR, suggests that the sparsity ratio and the number of known interactions may affect performance. Nonetheless, the overall performance is good even though the sparsity ratio is high since DTi2Vec can effectively deal with sparsity problems and imbalance issues. Figure 4 illustrates the connection between the sparsity ratio and the performances in terms of AUPR applied in two different classifiers.

### Comparing the performances of DTi2Vec and the state-of-the-art methods

Here we compare DTi2Vec's performance on the NR, GPCR, IC, E, and FDA_DrugBank datasets with five state-of-the-art methods, which include DTiGEMS + [28], TriModel [37], DDR [27], DNLMF [43], and NRLMF [45]. We selected these methods to give a comprehensive comparison and a broad perspective of our method prediction performance against the most representative and successful network-based DTIs prediction methods. Since DTiGEMS+ [28] is more recent and is the extension method of DTiGEM [29] that achieved better results, we exclude the latter from the comparison.

Table 4 shows DTi2Vec outperforms all the state-of-the-art methods based on it obtaining the highest averageAUPR for the four Yemanashi_08 datasets. It also obtained the highest averageAUPR for all datasets, including the FDA_DrugBank dataset, which is 3% higher than the second-best method (DTiGEMS+) in the first case and 2% higher than the third-best method (DTiGEMS+) in the second case. It also achieved the best averageAUC in the two cases. However, since the AUC results are already high in most other methods, DTi2Vec only showed a slight improvement in this metric (see Additional file 1: Table S2), but this is not the case for the AUPR performance metric (see Table 4). We separated the calculation of averageAUPR to have two averages, one across Yamanashi_08 datasets and the other across all datasets, which includes the FDA_DrugBank dataset, to show our method's high performance in both cases. We applied this evaluation because most of the current methods have not achieved high performance using FDA_DrugBank in terms of AUPR, and we show our method performs better with and even without the FDA_DrugBank dataset. Table 4 shows the best and second-best results in each category.

**Table 4:** Prediction performances for DTi2Vec and all comparison methods across all benchmark datasets

Dataset	Metric	Method					
		NRLMF	DNILMF	DDR	TriModel	DTiGEMS+	DTi2Vec
*Yamanishi_08 datasets*	***AvgAUPR***	0.80	0.78	0.87	0.88	0.92	**0.95**
	*AvgAUC*	0.95	0.95	0.96	0.98	0.99	**1.00**
*All datasets* *(Yamanishi_08 and FDA_DrugBank)*	***AvgAUPR***	0.72	0.69	0.82	0.84	0.86	**0.93**
	*AvgAUC*	0.94	0.95	0.96	0.98	0.99	**0.99**
*FDA_DrugBank* *(Hold-out test set)*	***AUPR***	0.34	0.31	0.63	0.66	0.62	**0.82**
	*AUC*	0.93	0.95	0.97	**0.99**	0.97	**0.99**

We rounded off all results to two decimal places. The bold underlined font indicates the best result in each category, while underlined values indicate the second-best outcome

**Fig. 5:**
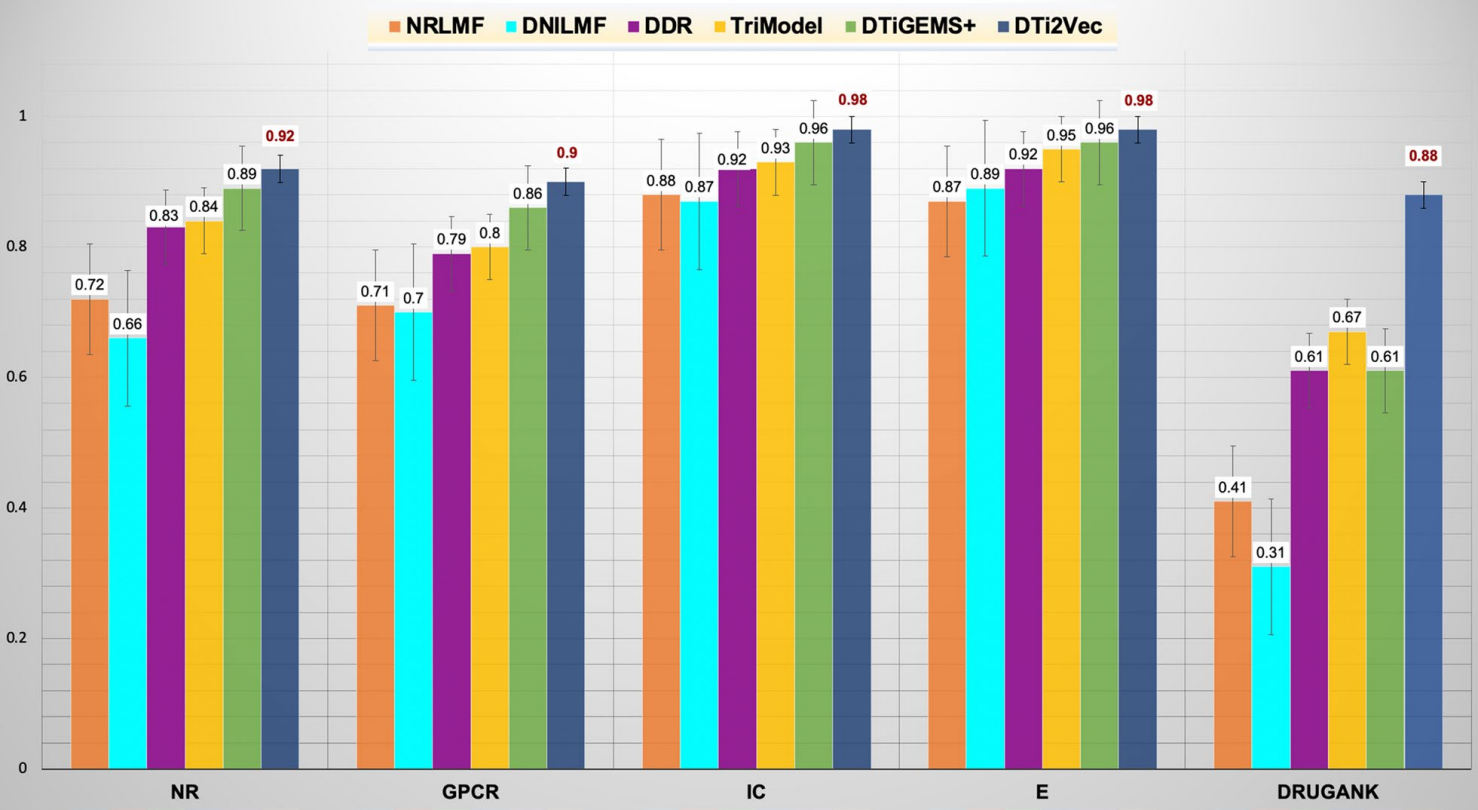
Comparing the prediction performance of DTi2Vec and state-of-the-art methods in random CV setting (in terms of AUPR with standard errors are shown) using the Yamanishi_08 and FDA_DrugBank datasets

As shown in Fig. 5, DTi2Vec outperformed the other state-of-the-art methods in terms of AUPR 0.92, 0.90, 0.98, 0.98, 0.88 for the NR, GPCR, IC, Enzyme, and FDA_DrugBank datasets, respectively. Additional file 1: Table S3 provides each fold result for 10-folds CV with the standard deviation of AUPR in 10-folds CV for each dataset (to show the stability of the results). For the NR, GPCR, IC, and Enzyme datasets, DTi2Vec outperformed the second-best method (DTiGEMS+) by 3%, 4%, 2%, and 1%, respectively. For the FDA_DrugBank dataset, DTi2Vec outperformed the second-best TriModel by 21%. Furthermore, to show that our results are statistically significant compared to the other methods, we applied the Wilcoxon test [67, 68] (a nonparametric statistical test that compares two paired groups). The Wilcoxon test comes in two versions, the Signed Rank test or the Rank Sum test, based on the individual AUPR values for each fold in the tenfold CV (see Additional file 1: Table S3). We demonstrate that the DTi2Vec results reflect an increase in the performance that is statistically significant compared to the second-best method (DTiGEMS + , TriModel) with probability values (p-values) < 0.05 obtained over GPCR, IC, E, and FDA_DrugBank datasets as 0.021, 0.014, 0.001 and 0.0002, respectively, except for the NR dataset which has p-value > 0.05.

We calculated the AUPR score error rate (ER) and the relative reduction of the AUPR score error rate of the best method (DTi2Vec) relative to the second-best method, defined previously in Eqs. 4 and 5, respectively, to show DTi2Vec's performance improvement and its robustness. Using obtained AUPR of tenfold CV experiments, the ER = 8%, 10%, 2%, 2%, and 12% for NR, GPCR, IC, E, and FDA_DrugBank, respectively. For predicting DTIs in NR, GPCR, IC, and Enzyme, our method DTi2Vec significantly reduces AUPR error relative to the next best method (DTiGEMS+) by 33%, 29%, 50%, and 33%, respectively. For the FDA_DrugBank dataset, the relative reduction of the AUPR error obtained by DTi2Vec relative to the next best method (TriModel) is 63%. Our method, DTi2Vec, consistently reduced the relative error rate compared to the state-of-the-art methods.

Nonetheless, we performed one more experiment using hold-out test data to demonstrate DTi2Vec's prediction reliability. We used the FDA_DrugBank dataset and split the data into three sets for training, validation, and testing. First, we initialized the hyperparameter values using the values obtained in the optimization process for the first experiment. Then we generated node2vec embeddings and used these embeddings to train the model. Next, we evaluated DTi2Vec on the validation set. We repeated this process several times to test random hyperparameter values for tuning, similar to Randomized Search. Finally, we generated embeddings using the optimized hyperparameter set and evaluated the model prediction performance on the test set. Using the random prediction setting, DTi2Vec achieves the highest AUPR on the hold-out test data compared to state-of-the-art methods (Table 4). Relative to the second-best method (TriModel), DTi2Vec reduces the AUPR error rate for the FDA_DrugBank dataset by 47%.

**Fig. 6:**
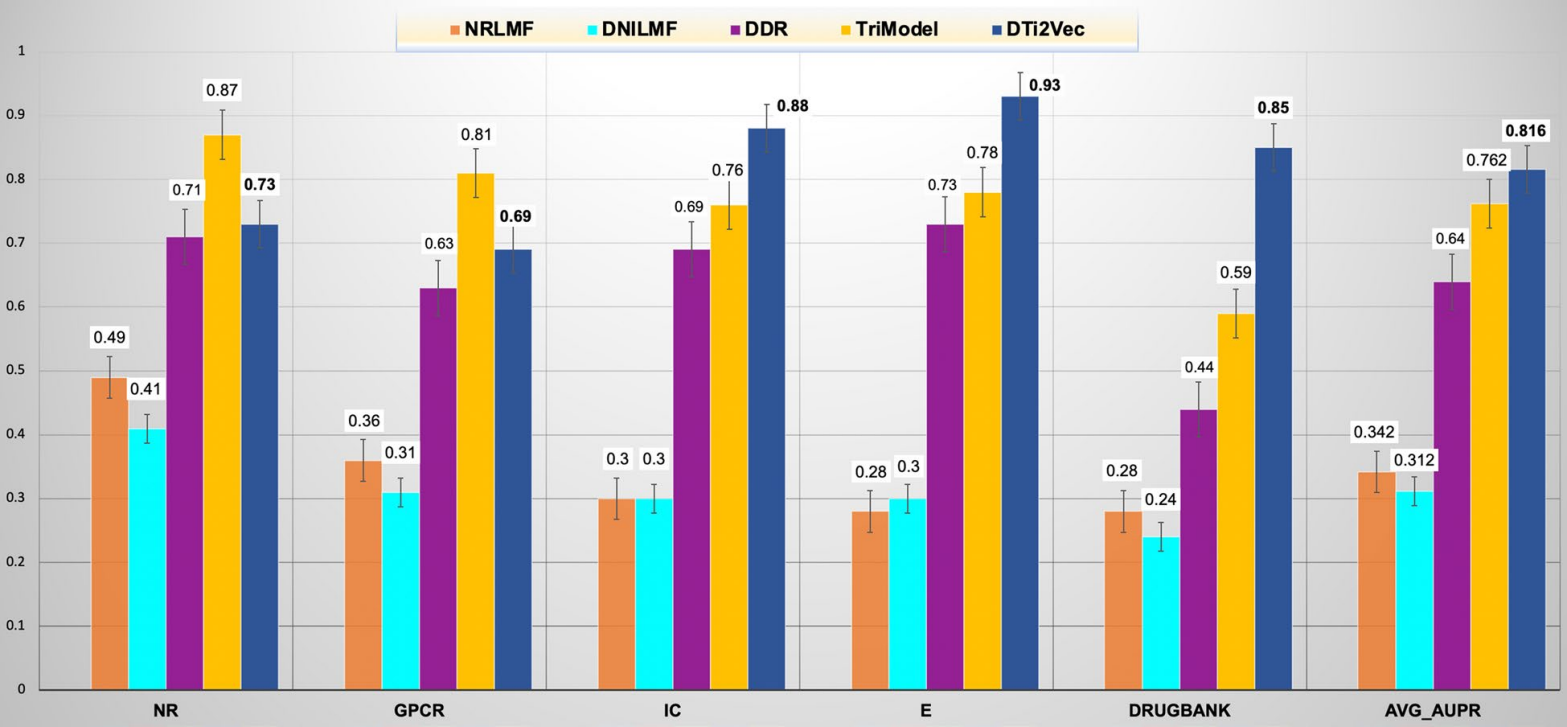
Comparing the prediction performance of DTi2Vec and other state-of-the-art methods when using new drug settings (in terms of AUPR using the Yamanishi_08 and FDA_DrugBank datasets, and the average AUPR across all datasets)

For the other experimental setting, when DTi2Vec predicted interactions for the new drugs, we obtained the results using all sets of FVs generated using the fusion functions except for the AVG function that always performed the worst, and we provided these results in the Additional file 1: Table S4. In addition, we compared the results with the same state-of-the-art methods but excluding DTiGEMS+ since it does not handle new drug settings. The best-obtained results when predicting new drugs are reflected in Fig. 6, which shows DTi2Vec outperformed all the state-of-the-art methods based on average AUPR across all datasets. Moreover, the significantly higher performance compared to the second-best (TriModel) is associated with the larger datasets, IC (AUPR 12%), Enzyme (AUPR 15%), and DrugBank (AUPR 26%). The new drug setting results for all the state-of-the-art methods are taken from [37].

The last evaluation process we performed to show the practicality of the predictive power of DTi2Vec in real scenarios by establishing the ability of our model to reposition a specific drug other than a hub drug node. This ability is important as hub nodes will likely not be the subject of drug research and development as they are usually well-studied. We apply this assessment: first, we calculate the average precision for DTI prediction at each drug. Second, we average this value (i.e., the average precision) over 10-folds. Finally, we calculate the mean average precision (MAP) [69] as the mean of 10-folds average precision for each drug across all drug nodes in the graph. We show that DTi2Vec achieves high MAP values, over NR, GPCR, IC, E, and FDA_DrugBank datasets as 0.96, 0.82, 0.91, 0.91, and 0.72 respectively. We conclude that the hub nodes do not likely drive our model's overall performance from these results.

In summary and based on all reported results, DTi2Vec outperformed all state-of-the-art methods in two independent datasets: Yamanashi_08 datasets and the FDA_DrugBank dataset and proved its general applicability, and declared its effectiveness in the prediction of DTIs.

### Novel DTI prediction and validation

To further demonstrate and analyze the capability of DTi2Vec, we performed a complimentary evaluation of its ability to predict new DTIs for each dataset and then validate the DTIs. To predict the new DTIs, we used all positive samples to train the model and divided the negative samples into training and test sets. The predicted DTIs (unknown in the original data but predicted to be positive) with high scores in the testing data are ranked based on their scores. We report the top 5 ranked DTI for each dataset separately. We manually confirmed those novel candidate DTIs using scientific literature and biomedical databases, including DrugBank [70], KEGG [47], ChEMBL [71], PubChem [72], Comparative Toxicogenomics Database (CTD) [73], MATADOR and SuperTarget [50], and Uniprot knowledgebase [74]. Table 5 shows the top 5 ranked novel DTIs for Yamanishi_08 and FDA_DrugBank datasets with the validation evidence. When we did not find any evidence, we marked the evidence as unknown since there is no confirmation that this interaction exists. We confirmed 21 out of 25 (84%) of the newly predicted DTIs as known interaction (i.e., this includes the top 5 ranked DTI for each of the five datasets used). Here, it is important to note that the DTIs data was last updated in 2008; this may be why we managed to confirm so many of the novel DTIs.

**Table 5:** The highly ranked (top-5) candidate novel DTIs obtained by DTi2Vec for each dataset verified with evidence from databases and published literature

#	DrugID	Drug name	TargetID	Target name	Validation evidence
*NR dataset (KEGG IDs)*					
1	D00690	Mometasone furoate	hsa2908	NR3C1 (Glucocorticoid receptor)	PMID:8439518
2	D00075	Testosterone	hsa5241	PGR (Progesterone Receptor)	PMID: 23229004 PMID: 23933754 C: 386630
3	D00554	Ethinyl estradiol	hsa2100	ESR2 (Estrogen Receptor 2)	CTD: D004997
4	D00327	Fluoxymesterone	hsa5241	PGR (Progesterone Receptor)	*Unknown*
5	D00348	Isotretinoin	hsa5915	RARB (Retinoic acid receptor beta)	KG: D00348- DG01604, C
*GPCR dataset (KEGG IDs)*					
1	D05792	Salmeterol	hsa153	ADRB1 (adrenoceptor beta 1)	*Unknown*
2	D04625	Isoetharine	hsa154	ADRB2 (Adrenoceptor Alpha 1B)	PMID:21948594
3	D02358	Metoprolol	hsa154	ADRB2 (Adrenoceptor Alpha 1B)	DB:DB00264
4	D02250	Octreotide acetate	hsa6751	SSTR1 (Somatostatin Receptor 1)	CTD: D015282 PMID:16438887
5	D01712	Theophylline sodium acetate	hsa136	ADORA2B (adenosine A2b receptor)	KG: D01712
*IC dataset (KEGG IDs)*					
1	D02098	Proparacaine hydrochloride	hsa8645	KCNK5 (Potassium Two Pore Domain Channel Subfamily K Member 5)	*Unknown*
2	D00438	Nimodipine	hsa779	CACNA1S (Calcium Voltage-Gated Channel Subunit Alpha1S)	KG: D00438 DB:DB00393
3	D00649	Amiloride hydrochloride	hsa8911	CACNA1I (Calcium Voltage-Gated Channel Subunit Alpha1 I)	M: Amiloride *(direct)*
4	D00495	Paramethadione	hsa8913	CACNA1G (Voltage-dependent T-type calcium channel subunit alpha-1G)	KG: D00495 DB:DB00617*(indirect)*
5	D03365	Nicotine	hsa1137	CHRNA4 (Cholinergic Receptor Nicotinic Alpha 4 Subunit)	PMID: 17590520 KG: D03365 DB: DB00184
*Enzyme dataset (KEGG IDs)*					
1	D00542	Halothane	hsa1571	CYP2E1 (Cytochrome P450 2E1)	PMID:19442086
2	D00437	Nifedipine	hsa1559	CYP2C9 (Cytochrome P450 Family 2 Subfamily C Member 9)	CTD: D009543 PMID: 9929518
3	D00139	Methoxsalen	hsa1543	CYP1A1(Cytochrome P450 1A1)	PMID: 7702611
4	D00574	Aminoglutethimide	hsa1589	CYP21A2 (Cytochrome P450 Family 21 Subfamily A Member 2)	M: Aminoglutethimide *(indirect)* PMID: 8201961
5	D00410	Metyrapone	hsa1583	CYP11A1 (Cytochrome P450 Family 11 Subfamily A Member 1)	CTD: D008797
*FDA_DrugBank dataset (DrugBank IDs)*					
1	DB00801	Halazepam	Q9UN88	GABRQ (Gamma-aminobutyric acid receptor subunit theta)	DB, UP, C:3885575 KG: D00338
2	DB01012	Cinacalcet	O15530	PDPK1(3-phosphoinositide-dependent protein kinase 1)	*Unknown*
3	DB00424	Hyoscyamine	P08912	CHRM5 (Muscarinic acetylcholine receptor M5)	KG: D00147- DG00053
4	DB01589	Quazepam	P47870	GABRB2 (gamma-aminobutyric acid type A receptor subunit beta2)	KG: D00457
5	DB00546	Adinazolam	Q9UN88	GABRQ (gamma-aminobutyric acid type A receptor subunit theta)	DB, KG: D02770 -DG00911

*C* ChEMBL, *CTD* Comparative toxicogenomics database, *DB* DrugBank, *M* MATADOR, *KG* KEGG, *DG* chemical Drug Group from KEGG, *PMID* PubMed, *UP* UniProt Knowledgebase

### Key strength of DTi2Vec

This section highlights some reasons for improvements in DTi2Vec performance compared to the current state-of-the-art methods and how our results show high promise in identifying novel DTIs.

#### The advantages of DTi2Vec over DTiGEMS+ 

DTi2Vec is a simple yet effective method that achieves less error-prone results using one type of drug similarity and target/protein similarity augmented to DTIs. In contrast to other graph-based methods such as DDR and DTiGEMS+ , it leverages from the full weighted heterogeneous graph G, without the complexity of selecting and integrating multiple kernels (i.e., similarities) that require much effort and time. However, it can utilize different types of similarity that can be augmented to the full graph G and then follow the same steps.

DTi2Vec has several advantages over our previous method (DTiGEMS+ , the second-best method) using the Yamanishi_08 datasets, even though they have some similarities in the feature extraction pipeline, including applying the node2vec technique. DTi2Vec has an advantage over DTiGEMS+ in five major points:

- DTi2Vec performed much better in the most extensive dataset DrugBank, by increasing the prediction performance by 27% compared to DTiGEMS+ in terms of AUPR.
- DTi2Vec has a more straightforward pipeline with fewer steps but is more effective, faster, and require less memory than DTiGEMS+ mainly because:
- DTi2Vec constructs just one graph to auto-generate features for each drug–target edge, while DTiGEMS + constructed three graphs for the hand-crafted feature extraction for each drug–target pair.
- DTi2Vec utilized single drug–drug similarity and target–target similarity while DTiGEMS + calculated and utilized multiple drug–drug and target–target similarities then applied similarity selection and integration. All these steps increase the time and memory complexities.
- Both DTi2Vec and DTiGEMS + applied node2vec on the whole heterogeneous DTIs network, but they used the embeddings for each node in different ways. The DTi2Vec method takes advantage of the automatically learned feature representations in low dimensional space for each node. It directly applies the fusion function to create features for each drug–target pair. On the other hand, DTiGEMS + calculated the cosine similarity for each drug–drug and target–target pair separately to formulate a new heterogeneous network, from which the path score features are obtained, resulting in losing some valuable features directly related to some drug–target edges.
- DTi2Vec FVs are the drug–target edge embeddings, while DTiGEMS + FVs are the sum and max path-scores for each drug–target edge under six path structures. This difference can lead to the best characteristic of DTi2Vec is that the FVs are not sparse contrary to the situation in DTiGEMS + , where the FVs can be sparse. The reason is that DTi2Vec leverages the node2vec embeddings to directly construct edge representation using fusion function, resulting in converting the sparse graph data into low dimensional vector spaces used as features. In contrast, DTiGEMS + used node2vec embeddings to calculate new cosine similarity and used these similarities to construct a new heterogeneous graph to obtain path score features. Although applying graph mining to extract path score features between each drug–target pair provides meaningful features for the classifier in DTiGEMS + , it can suffer from the sparsity issue, specifically when the number of known DTIs is limited, which affects the classifier’s performance.
- The last advantage of DTi2Vec over DTiGEMS is that DTi2Vec can handle the newDrug setting by predicting novel interactions for new drugs, and it achieved good results.

#### Effectiveness of feature representation used with the heterogeneous network

Most of the prior network-based methods have focused on a homogeneous network (by using separately the drug similarity graph and target similarity graph). Each node obtains the features from the corresponding subgraph and then predicts the edge connecting these two graphs by inferring DTI. Considering only the homogenous graph might result in losing some informative links and meaningful features that enhance the DTIs prediction accuracy. DTi2Vec takes advantage of combining two techniques of working on a complete heterogeneous DTIs graph with graph embedding (i.e., representation learning) that provides a powerful graph analytical approach to capture meaningful and rich features. Specifically, we constructed the full, rich, heterogeneous DTIs network augmented with KNN drug similarity and targets similarity graphs. We then apply the node2vec model to learn an embedding (i.e., feature representation) for each node (drug and target). These generated representations incorporate the node topological context and structural information so that similar nodes will have similar numerical representations. Node representations are learned via the Skip-gram model by optimizing the likelihood objective using stochastic gradient descent (SGD). Then, an efficient representation is generated for each drug–target edge using several fusion functions.

#### Efficient performance in large scale data

Many recent DTI prediction methods have performed well in terms of AUPR evaluation metric using Yamanishi_8 datasets, but none have achieved good AUPR using the approved FDA_DrugBank dataset. The possible reason for this may be because the previous methods did not consider the feature representation for drug–target edges (and did not distinguish between the positive and negative edges (i.e., unknown interaction)). DTi2Vec overcame this limitation and achieved improved predictive performance by obtaining high AUPR (0.88) in the FDA_DrugBank dataset.

#### Effectiveness of the gradient boosting classifier

Although DL using deep neural network (DNN) demonstrated its efficiency in prediction problems and outperformed most other ML classifiers, we believe that DNN doesn't perform well in the classification stage using the FVs of all drug–target pairs in our work. We tested our hypothesis by applying a sequential DL model in FVs for each dataset, and the results were less than the results obtained using XGBoost. The result suggests DNN works better dealing with unstructured data such as images, text (e.g., protein sequences or drug SMILES in DTIs prediction problem) and when using very large data, which is not the case for the Yamanishi_08 datasets. At the same time, ensemble-based algorithms (including boosting classifiers: AdaBoost and XGBoost) perform better in structured/tabular data. We selected XGBoost as the primary classifier in our work, a scalable and accurate gradient boosting machine. XGBoost classifier was developed and optimized for overall performance and computational speed. It has demonstrated the ability to push the boundaries of computing power for boosted trees algorithms. Furthermore, we compare XGBoost to several ML ensemble classifiers and the forward neural network classifier (FNN) but did not include the results in this manuscript since AdaBoost and XGBoost outperformed them all. Several of the previous DTIs prediction methods have applied different versions of the ensemble/boosting classifiers, but none of them leverage the advantage of using the XGBoost classifier.

## Conclusion

This work has developed an efficient computational method that solved DTI identification as a link prediction problem in heterogeneous networks. This method, DTi2Vec, takes advantage of the heterogeneous network to infer the interaction instead of separately using each homogeneous graph. It integrated the drug similarity graph, target similarity graph, and known DTIs graph to formulate the complete weighted heterogeneous graph *G(V, E)*, a more informative network to enhance the prediction performance. The Node2vec model was applied to this graph *G*, to generate efficient feature representations for each node and then learn feature representation for each drug–target edge using different fusion functions. These features proved to be very useful and informative, capturing all needed information from the graph for DTI prediction. It is generated automatically by omitting time-consuming feature engineering steps that can potentially affect prediction accuracy. We fed the FVs to two ensemble classifiers for a better comparison of prediction performance. DTi2Vec significantly increases the prediction performance compared to five state-of-the-art methods using Yamanishi_08 and FDA_DrugBank datasets and multiple evaluation metrics, which indicates the robustness of DTi2Vec. Furthermore, DTi2Vec demonstrates its efficiency in the reliability of results (based on the AUPR), and predicting new DTIs, validated using several databases and published literature.

One limitation worth mentioning is that DTi2Vec cannot predict the interaction of new targets or newDrug-newTarget pairs. We intend to overcome this limitation in future work by extending DTi2Vec capabilities to deal with other settings, including new target settings or newDrug-newTarget pairs, beyond the commonly applied random setting. As future work, we also plan to further improve the performance by applying and utilizing different representation learning algorithms (i.e., graph embedding techniques) and using different types of similarity of drug-drug and target-target that may be more informative. We also want to use our model to develop a real-life case study related to drug repositioning in cancer and experimentally validate our model's selected predictions to demonstrate our results' clinical relevance. Another extension of our method will be formulating the task as a regression model to predict the drugs and target binding ability that reflects more meaningful interaction. The last significant extension is that our network-based method is suitable for DTIs prediction, but it can be generalized to any network-based problem in biomedical domains such as drug-drug interactions network, drug-disease indication network, protein-disease association network, and others.

## Supplementary Information

**Additional file 1: Table S1.** Tested Values (recommended by node2vec [1]) and the optimal parameter values for each dataset. **Table S2.** The AUC scores for all methods for each dataset separately. All results are rounded to 2 digits. Bold fonts with underline indicate the best results while bold fonts indicate the second-best. **Table S3.** DTi2Vec best performance for each dataset in terms of AUPR, in each fold of 10-folds CV, AvgAUPR, standard deviation (std), and p-values. **Table S4.** Performance of DTi2Vec in terms of AUPR using XGBoost classifier on each dataset with multiple FVs generated by applying different edge representation functions for new drug setting. Bold underlined font indicates the best result in each dataset.

## Data Availability

All datasets and the source code used in this study are available in the following GitHub link: https://github.com/MahaThafar/DTi2Vec.

## Abbreviations

Adaboost: Adaptive boosting
AUC: Area under ROC curve
AUPR: Area under the precision-recall curve
CART: Classification and Regression Trees
CV: Cross validation
DD sim: Drug–drug similarity matrix
DL: Deep learning
DTIs: Drug–target interactions
E: Enzyme
ER: Error rate
FDA: Food and Drug Administration
FV: Feature vectors
FPR: False positive rate
GPCR: G-protein-coupled receptor
IC: Ion channel
KG: Knowledge graph
KNN: K-nearest neighbor
MAP: Mean average precision
MF: Matrix factorization
ML: Machine learning
MLP: Multilayer Perceptron
NBI: Network-based inference
NLP: Natural language processing
NN: Neural network
NR: Nuclear receptor
PSM: Path score model
P-value: Probability value
ROC: Receiver operating characteristic
TPR: True positive rate
TT sim: Target-target similarity matrix
XGBoost: EXtreme Gradient Boosting
