# Supplementary material for: DTi2Vec: Drug–target interaction prediction using network embedding and ensemble learning
Source: J Cheminform. 2021 Sep 22;13:71. doi: 10.1186/s13321-021-00552-w (PMC8459562; doi:10.1186/s13321-021-00552-w)
Supplement: Supplementary file 1 — Additional file 1: Table S1. Tested Values (recommended by node2vec [1]) and the optimal parameter values for each dataset. Table S2. The AUC scores for all methods for each dataset separately. All results are rounded to 2 digits. Bold fonts with underline indicate the best results while bold fonts indicate the second-best. Table S3. DTi2Vec best performance for each dataset in terms of AUPR, in each fold of 10-folds CV, AvgAUPR, standard deviation (std), and p-values. Table S4. Performance of DTi2Vec in terms of AUPR using XGBoost classifier on each dataset with multiple FVs generated by applying different edge representation functions for new drug setting. Bold underlined font indicates the best result in each dataset. [file 13321_2021_552_MOESM1_ESM.docx]

**DTi2Vec: Drug-Target Interaction Prediction using network embedding and ensemble learning**

Maha A. Thafar^1,2^, Rawan S. Olayan ^3^, Somayah Albaradei^1,4^, Vladimir B. Bajic^1^, Takashi Gojobori^1^, Magbubah Essack^1*^, Xin Gao^1*^

**Additional Materials**

**Table S1: Tested Values (recommended by node2vec [1]) and the optimal parameter values for each dataset.**

| **node2vec Parameters** | **NR** | **GPCR** | **IC** | **Enzyme** | **FDA_DrugBank** |
| --- | --- | --- | --- | --- | --- |
| **dimensions --d**  Tested values: [32, 64, 128, 256, 512, 1024] | 32 | 64 | 64 | 32 | 128 |
| **--walk-length**  Tested values: is different from one dataset to another based on the dataset size | 50 | 80 | 100 | 150 | 400 |
| **Return hyperparameter -- p**  Tested values: {0.25, 0.5, 1, 2, 4} | 0.25 | 0.5 | 0.5 | 1 | 1 |
| **In-out hyperparameter --q**  Tested values: {0.25, 0.5, 1, 2, 4} | 0.5 | 2 | 0.25 | 1 | 2 |
| **--num-walk**  Tested values: [5, 10, 20] | 10 | 10 | 10 | 10 | 10 |

**Table S2.** The AUC scores for all methods for each dataset separately. All results are rounded to 2 digits. Bold fonts with underline indicate the best results while bold fonts indicate the second-best.

| **Dataset** | **AUC of each method** | | | | | |
| --- | --- | --- | --- | --- | --- | --- |
|  | NRLMF | DNILMF | DDR | TriModel | DTiGEMS+ | DTi2Vec |
| **NR** | 0.93 | 0.92 | 0.975 | **0.97** | **0.97** | **0.99** |
| **GPCR** | 0.95 | 0.96 | 0.984 | **0.98** | **0.99** | **0.99** |
| **IC** | 0.98 | 0.94 | 0.982 | **0.99** | **0.99** | **1.00** |
| **Enzyme** | 0.95 | 0.96 | 0.991 | **0.99** | **0.99** | **1.00** |
| **FDA_DrugBank** | 0.93 | 0.95 | 0.971 | **0.99** | 0.98 | **0.99** |

**Table S3: DTi2Vec best performance for each dataset in terms of AUPR,** in each fold of 10-folds CV, AvgAUPR, standard deviation (std), and p-values.

| **Dataset** | **NR** | **GPCR** | **IC** | **Enzyme** | **FDA_DrugBank** |
| --- | --- | --- | --- | --- | --- |
| **Fusion Function**  **Classifier** | WL1  AdaBoost | Hadamard  XGBoost | Concatenate  XGBoost | Concatenate  XGBoost | Hadamard  XGBoost |
| fold-1 | 0.941 | 0.815 | 0.975 | 0.986 | 0.871 |
| fold-2 | 0.933 | 0.892 | 0.984 | 0.985 | 0.873 |
| fold-3 | 0.804 | 0.955 | 0.982 | 0.985 | 0.879 |
| fold-4 | 1.00 | 0.864 | 0.991 | 0.971 | 0.868 |
| fold-5 | 0.924 | 0.936 | 0.971 | 0.969 | 0.884 |
| fold-6 | 0.975 | 0.931 | 0.970 | 0.978 | 0.899 |
| fold-7 | 1.00 | 0.881 | 0.985 | 0.972 | 0.876 |
| fold-8 | 0.781 | 0.904 | 0.966 | 0.976 | 0.880 |
| fold-9 | 0.853 | 0.920 | 0.964 | 0.978 | 0.889 |
| fold-10 | 1.00 | 0.90 | 0.966 | 0.988 | 0.882 |
| **AvgAUPR** | **0.92** | **0.90** | **0.98** | **0.98** | **0.88** |
| **std** | 0.08 | 0.038 | 0.0089 | 0.0065 | 0.0087 |
| **p-values** | 0.25 | 0.021 | 0.014 | 0.001 | 0.0002 |

**Table S4: Performance of DTi2Vec in terms of AUPR using XGBoost classifier on each dataset with multiple FVs generated by applying different edge representation functions for new drug setting.** *Bold underlined font indicates the best result in each dataset.*

| Datasets /  Fusion Function for FV | NR | GPCR | IC | Enzyme | DrugBank |
| --- | --- | --- | --- | --- | --- |
| Concatenate | 0.68 | **0.69** | 0.81 | 0.85 | 0.74 |
| Hadamard | 0.72 | 0.61 | 0.87 | **0.93** | **0.85** |
| Weighted L1 | **0.73** | **0.69** | **0.88** | **0.93** | 0.83 |
| Weighted L2 | 0.72 | 0.68 | **0.88** | 0.92 | 0.83 |

**References:**

1. Grover, A. and J. Leskovec, *node2vec: Scalable Feature Learning for Networks.* KDD, 2016. **2016**: p. 855-864.
